# An imine reductase that captures reactive intermediates in the biosynthesis of the indolocarbazole reductasporine

**DOI:** 10.1016/j.jbc.2024.105642

**Published:** 2024-01-08

**Authors:** Phillip Daniel-Ivad, Katherine S. Ryan

**Affiliations:** Department of Chemistry, The University of British Columbia, Vancouver, British Columbia, Canada

**Keywords:** imine reductase, indolocarbazole, biosynthesis, x-ray crystallography, enzyme structure, enzyme catalysis, site-directed mutagenesis

## Abstract

Imine reductases (IREDs) and reductive aminases have been used in the synthesis of chiral amine products for drug manufacturing; however, little is known about their biological contexts. Here we employ structural studies and site-directed mutagenesis to interrogate the mechanism of the IRED RedE from the biosynthetic pathway to the indolocarbazole natural product reductasporine. Cocrystal structures with the substrate-mimic arcyriaflavin A reveal an extended active site cleft capable of binding two indolocarbazole molecules. Site-directed mutagenesis of a conserved aspartate in the primary binding site reveals a new role for this residue in anchoring the substrate above the NADPH cofactor. Variants targeting the secondary binding site greatly reduce catalytic efficiency, while accumulating oxidized side-products. As indolocarbazole biosynthetic intermediates are susceptible to spontaneous oxidation, we propose the secondary site acts to protect against autooxidation, and the primary site drives catalysis through precise substrate orientation and desolvation effects. The structure of RedE with its extended active site can be the starting point as a new scaffold for engineering IREDs and reductive aminases to intercept large substrates relevant to industrial applications.

Imine reductases (IREDs) (1, 2) have drawn considerable interest for their application as biocatalysts in the synthesis of pharmaceuticals (3, 4, 5, 6). IREDs can be used in chiral amine syntheses starting from imines (7, 8) or amine-ketone pairs (9). They are valued for their ability to create secondary and tertiary chiral amines, as well as catalyzing reductive amination (6, 10). Recently, two reductive aminases (RedAms) have been engineered to produce industrially useful amounts of drug candidates (11, 12). However, with the focus on industrial applications and engineering of IREDs, the study of these enzymes in their natural context has been neglected.

RedE, an IRED, serves as a biosynthetic enzyme in the formation of the indolocarbazole natural product reductasporine (13). Indolocarbazoles have been a rich source of potential chemotherapeutics with many entering clinical trials for activity against cancers (14), and the semisynthetic staurosporine derivative, midostaurin, was approved in 2017 as an anticancer treatment (15). In the field of biosynthesis, a number of studies have highlighted the shared features of indolocarbazole assembly, with initial steps catalyzed by a core set of oxidative enzymes, followed by the action of diverse tailoring enzymes (16). Each indolocarbazole gene cluster preserves three genes coding for an FAD-dependent tryptophan oxidase (*redO*, *rebO*, and *staO*), a heme-containing chromopyrrolic acid (CPA) synthase (*redD*, *rebD*, and *staD*) and a cytochrome P450 indolocarbazole synthase (*redP*, *rebP*, and *staP*) (Fig. S1). The tryptophan oxidase first converts two l-tryptophan molecules to the corresponding imines before CPA synthase couples the two imines at the C*β* position (17). The indole-3-pyruvate imine dimer spontaneously cyclizes into CPA (18, 19). The indolocarbazole synthase oxidatively bridges the indole C2 positions yielding a proposed dicarboxy indolocarbazole intermediate (4) (20, 21). This indolocarbazole is prone to decarboxylation and oxidation and has not yet been directly detected. Several indolocarbazole biosynthetic gene clusters encode unique enzymes that capture and stabilize an indolocarbazole decomposition product before promoting the formation of a more stable compound for further tailoring (22, 23, 24, 25, 26). For example, the rebeccamycin biosynthetic enzyme RebC and the homologous staurosporine biosynthetic enzyme StaC capture reactive intermediates and funnel them toward the 8- and 4-electron oxidation products, respectively (Fig. S1*B*). The trapping of distinct tautomers of 7-carboxy-K252c in crystals of RebC and in RebC-10x, an enzyme with StaC-like activity, suggest that careful control of the hybridization at the C7 position can enable simple decarboxylation in the keto tautomer to staurosporine aglycone K252c (in the case of StaC) or decarboxylation after hydroxylation of the enol tautomer to arcyriaflavin A (AFA) (in the case of RebC) (23, 25).

The reductasporine gene cluster was discovered while screening the metagenomes of soil samples for CPA synthase-like genes (13). Chang *et al.* reconstituted this pathway in *Escherichia coli* and proposed RedE reduces the 5,7-didehydro-N,N-didemethylreductasporine (**3**) to didemethylreductasporine (**2**) (Fig. 1). This proposal is based on the propensity of the dicarboxyindolocarbazole intermediate **4** to spontaneously decarboxylate at physiological conditions, although **3** was not detected directly (13, 20). Recently, our group reconstituted reductasporine biosynthesis *in vitro* (27), reproducing the RedE-dependent formation of **2** from indolocarbazole synthase products and subsequent methylation *via* RedM to reductasporine (**1**). In analogy to the role of the StaC/RebC enzymes, RedE captures a reactive intermediate from the indolocarbazole biosynthetic pathway; however, it catalyzes a reduction rather than an oxidation. Based on these biochemical data, RedE would have to intercept an intermediate while not allowing for oxidation, indicating a novel mechanism.

**Figure 1:**
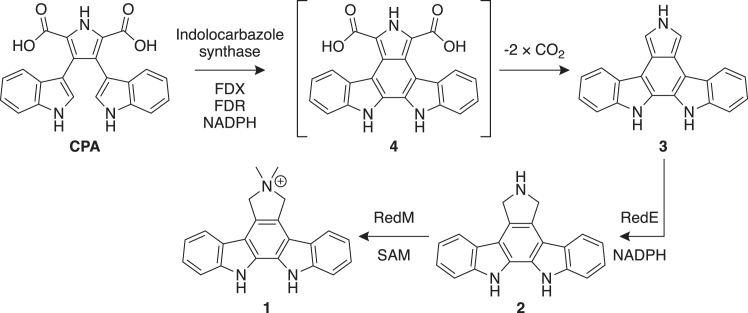
**Proposed biosynthetic pathway to reductasporine (1).** RedE intercepts spontaneous decarboxylation products after a cytochrome P450 indolocarbazole synthase catalyzes the bridging of the C2 positions from each indole in CPA. The simplest substrate for RedE is 5,7-didehydro-N,N-didemethylreductasporine (**3**), which is proposed to be reduced to didemethylreductasporine (**2**) before methylation to **1***via* SAM-dependent methyltransferase, RedM. The indolocarbazole synthase requires coenzymes ferredoxin (FDX) and ferredoxin reductase (FDR) powered by NAD(P)H for activity. CPA, chromopyrrolic acid; SAM, S-adenosyl methionine.

Here, we present crystal structures of RedE, including apo, NADP^+^-bound and two ternary complexes with AFA and NADP(H). These crystal structures reveal an unexpected secondary indolocarbazole binding site adjoined to the primary active site, and site-directed mutagenesis shows both are critical for catalysis. Our biochemical characterization of StaP reaction products reveals the accumulation and decomposition of 3, and we propose that RedE intercepts and reduces **3** from solution. The secondary active site may protect the indolocarbazole from oxidation and select for the catalytically compatible isoindole tautomer before direct transfer to the primary site for reduction. Close inspection of the secondary indolocarbazole binding site and comparison to other structurally characterized IREDs suggest potential sequence determinants that would favor the formation of this cleft, indicating wider implications on the malleability of IRED active sites.

## Results

### RedE crystal diffraction dataset phasing, twinning, and initial model building

**Figure 2:**
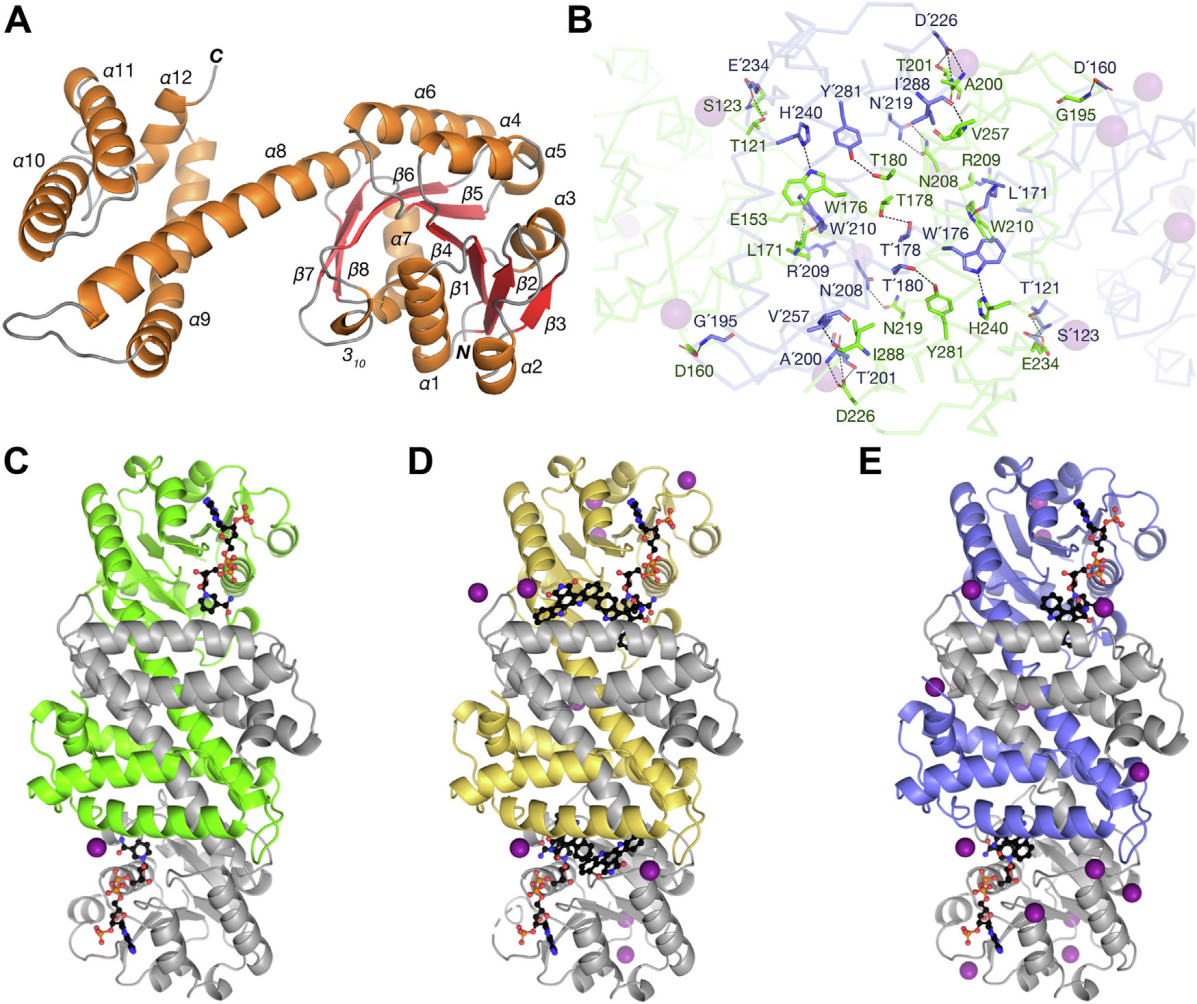
**Cartoon representations of the overall RedE structure.***A*, protomer with highlighted secondary structures. A 3_10_-helix forms between *β*6 and *β*7. *B*, polar contacts between protomers of the RedE dimer. Asymmetric units of the (*C*) RedE-NADP^+^ binary complex, (*D*) RedE-AFA complex copurified with NADP(H), and (*E*) RedE-AFA-NADP^+^ ternary complex. Ligand molecules shown as ball-and-stick models in *black*, and iodide ions as *purple spheres*. AFA, arcyriaflavin A.

We solved the apo, binary complex and two ternary complex structures of RedE with NADP^+^ and AFA to resolutions of 1.60 to 1.63 Å (Table 1, Fig. 2). The diffraction dataset phases for these models were solved *via* molecular replacement using a selenomethionine-substituted RedE model (SeMet-RedE), which was itself phased *via* single anomalous dispersion of selenium atoms. Twinning was detected in SeMet-RedE and apo-RedE crystal datasets. The second moment of intensity (⟨I^2^⟩/⟨I⟩^2^) reaches 1.78 (untwinned: 2.0, perfect twin: 1.5) and average local intensity differences, ⟨|L|⟩, 0.43 (untwinned: 0.5, perfect twin: 0.375). The L-test for twinning identifies a twin fraction as high as 23%. When a monoclinic unit cell fulfills any condition *β* ≈ 90°, a ≈ c, or 2c·cos *β* ≈ −a it is possible to form a pseudomerohedral twin (28, 29, 30). The cell dimensions of the P 2_1_ space group crystals of SeMet-RedE and apo-RedE meet the third condition, and a model can be solved for using the appropriate twin law (h, −k, −h−l). The resulting SeMet-RedE model arranges as a pair of dimers in the asymmetric unit. In several chains, *α*11 (residues 256–278) has too poor electron density to model completely, and another 16 residues in several loop regions are also missing (Fig. S2). Nonetheless, this model is sufficient to solve for phases *via* molecular replacement in other RedE diffraction datasets. RedE cocrystals with NADP^+^ and AFA were not affected by twinning.

**Table 1:** Crystallographic data collection and refinement statistics^a^

	Apo-RedE	RedE-NADP^+^	RedE-AFA-NADP^+^	RedE-AFA
Wavelength (Å)	0.9796	0.9796	0.9796	0.9796
Resolution (Å)	93.36–1.62 (1.65–1.62)	68.88–1.62 (1.65–1.62)	37.75–1.60 (1.63–1.60)	37.94–1.63 (1.67–1.63)
Space group	P 2_1_	P 2_1_	P 2_1_	P 2_1_
Cell dimensions (Å)	51.78 186.72 71.69 *β* = 111.12°	44.74 111.31 72.04 *β* = 107.04°	54.11 110.77 66.99 *β* = 107.60°	54.59 111.53 67.69 *β* = 108.47°
R_merge_	0.095 (1.425)	0.080 (0.414)	0.073 (0.887)	0.071 (0.665)
R_meas_	0.103 (1.546)	0.087 (0.449)	0.078 (0.957)	0.077 (0.722)
R_pim_	0.040 (0.593)	0.034 (0.172)	0.029 (0.355)	0.030 (0.276)
Total number of reflections	999547 (50013)	493577 (27473)	677620 (32657)	644294 (34386)
Total number of unique reflections	154438 (7659)	75988 (4141)	95462 (4628)	93667 (5126)
⟨I/***σ***(I)⟩	11.9 (1.6)	12.9 (4.2)	14.4 (2.2)	12.8 (2.3)
CC_1/2_	0.998 (0.448)	0.996 (0.945)	0.999 (0.845)	0.998 (0.948)
Completeness	96.3 (97.6)	88.9 (97.9)	96.6 (95.1)	98.7 (97.9)
Multiplicity	6.5 (6.5)	6.5 (6.6)	7.1 (7.1)	6.9 (6.7)
Wilson B-factor	20.26	16.61	17.91	22.98
Reflections used in refinement	154374 (7727)	75899 (2967)	95375 (6731)	93448 (7688)
Reflections used for R_free_	7991 (408)	3872 (151)	2000 (141)	1708 (141)
R_work_	0.1633 (0.2777)	0.1715 (0.2139)	0.1553 (0.2574)	0.1929 (0.3384)
R_free_	0.2076 (0.2817)	0.2050 (0.2792)	0.1889 (0.2682)	0.2191 (0.3900)
Number of non-hydrogen atoms	9463	5005	5191	4894
macromolecules	8280	4160	4275	4180
ligands	33	140	154	128
solvent	1150	705	762	586
Protein residues	1152	578	581	566
RMS (bonds)	0.002	0.005	0.010	0.005
RMS (angles)	0.52	0.77	1.08	0.71
Ramachandran favored (%)	97.63	98.26	98.09	98.03
Ramachandran allowed (%)	2.28	1.74	1.91	1.97
Ramachandran outliers (%)	0.09	0.00	0.00	0.00
Rotamer outliers (%)	0.62	0.50	0.48	0.25
Clashscore	3.43	1.54	2.43	2.40
Average B-factor	23.46	22.89	26.33	34.41
macromolecules	21.10	20.45	23.77	32.36
ligands	36.51	28.54	28.48	43.59
solvent	40.07	36.17	40.27	47.01
Number of TLS groups	14	8	6	6

a Data in parentheses are for the high-resolution shell.

### Structure of RedE

RedE resembles the canonical IRED fold with an N-terminal NADPH-binding Rossmann domain and a C-terminal helical bundle mediating homodimerization (8, 9, 10, 31). The N-terminal Rossmann domain (residues 2–161) consists of an eight-stranded *β*-sheet with the first six strands (*β*1-6) running parallel and the following two strands (*β*7-8) antiparallel. Each parallel strand is bounded by an *α* helix (*α*1-7) forming *βαβ* motifs and a 3_10_-helix forms in the loop between the antiparallel *β*6 and *β*7 strands (Fig. 2*A*). The common Rossmann domain GxGxxG nucleotide binding motif is preserved at residues 9 to 14 (GLGPMG) (32). The C-terminal domain (residues 193–288) is a five-membered helical bundle where the latter four *α*-helices (*α*9-12) wrap around the interdomain helix of its dimer partner (*α*ʹ8) forming an extensive 4020 Å^2^ interface (27% of total monomer surface area) between both N- and C-terminal domains as calculated by PISA software (33). A total of 22 and 79 residues are involved in polar and hydrophobic contacts, respectively (Fig. 2*B*). The majority of these interactions are within the dimerization domains with only 17 residues interfacing between N- and C-terminal domains. Hydrogen bonding and electrostatic interactions occur between E153 to Rʹ209, D160 to Gʹ195, and T121 and S123 to Eʹ234. The N-terminal end of *α*6 is associated with an iodide ion which is in turn stabilized by Rʹ254. The majority of the hydrophobic contacts between the two domains are found between *β*6-8 and *α*ʹ9 where M119, Y130, L131, L133, M155, and L157 interlock with Vʹ206, Rʹ209, Wʹ210, and Tʹ213. Lastly, residues at the N-terminal ends of *α*6 and the interdomain helix (P96, V97, A162, and M163) stack against the loop between the C-terminal end of the dimer partner’s interdomain helix and *α*ʹ9, specifically Pʹ195 and Gʹ196.

**Figure 3:**
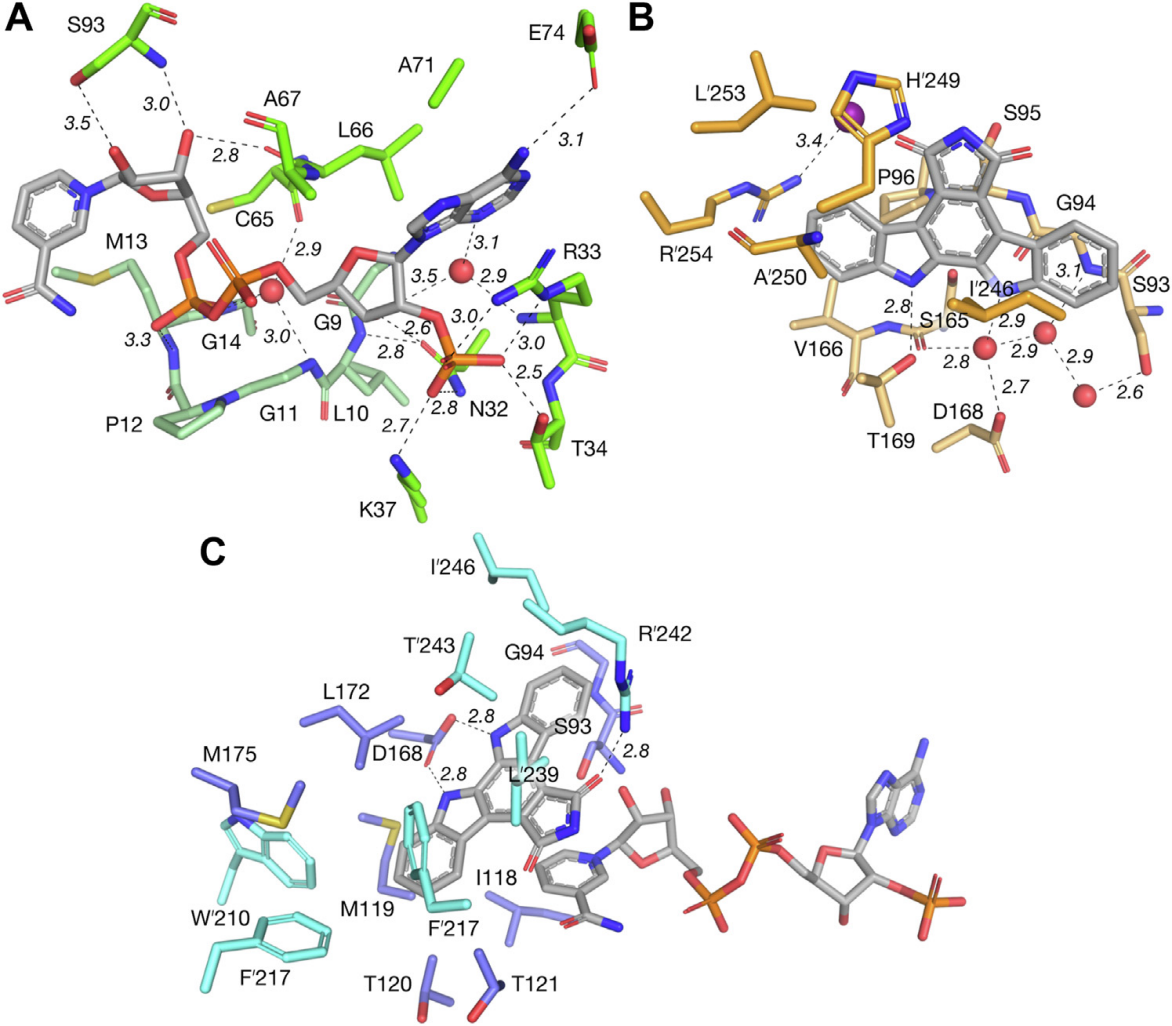
**Key interactions in the in the extended RedE active site.** Hydrogen bonds are indicated by *dashed lines* with distances labeled in Angstrom. *A*, the NADPH binding cleft of RedE. The conserved GxGxxG Rossmann domain binding motif is colored *pale green* (9-GLGPMG-14). *B*, the secondary indolocarbazole binding site occupied by arcyriaflavin A (AFA). Residues from the neighbouring protomer are colored *orange*. *C*, the primary indolocarbazole binding site occupied by AFA. The maleimide carbonyl is positioned 3 Å above the *si*-face of the nicotinamide ring in an ideal geometry for hydride transfer. Residues from the neighbouring protomer are colored *cyan*.

Datasets from crystals of RedE grown with 10 mM NADP^+^ produce F_o_–F_c_ electron density omit maps that can be unambiguously modelled as NADP^+^ (Fig. S3*A*). The cofactor binds a broad cleft formed from the confluence of *α*1, *α*2, and *β*1-5 with the characteristic GxGxxG NADPH binding sequence found at the center between *β*1 and *α*1 (9-GLGPMG-14) with hydrogen bonds forming between the C5ʹ-phosphate and M13 amide N-H and an ordered water held between the amide nitrogen atoms of G11 and G14 and C65 amide carbonyl (Fig. 3*A*). The adenosine C2ʹ-phosphate is held in place by a salt bridge with R33 alongside hydrogen bonds with Q32, T34, and K37. The adenine ring rests against the A71 side chain and is parallel stacked with the R33 guanidino group. E74 hydrogen bonds to the adenine C6-amine. The backbone N-H of A67 hydrogen bonds with the adenosine furan oxygen, while the S83 side chain and L66 backbone carbonyl and interact with the C2ʹ- and C3ʹ-hydroxyl of the nicotinamide riboside, respectively. The re-face of the nicotinamide C4 is positioned overtop the side chain of M13 with the si-face facing into a deep pocket formed at the dimer interface of the C-terminal helical bundle. Comparison of the apo and binary structures show that R33 moves out of the adenine binding area in order to stack against the purine ring while forming a salt bridge to the nearby 2ʹ-phosphate.

As the substrate and products of RedE are transiently stable, we elected to use AFA as a mimic, because it shares the planar indolocarbazole core of **3**. Processing diffraction data of RedE crystallized from solutions with 1.2 mM AFA and 10 mM NADP^+^ show electron density consistent with both AFA and NADP^+^ in each protomer (Fig. S3, *A* and *B*). In this structure, the positioning of NADP^+^ is similar to that observed in the binary complex. AFA sits above the *si*-face of the nicotinamide C4 with the maleimide carbonyl situated 3 Å away in a geometry ideal for hydride transfer. AFA is enveloped by a highly hydrophobic pocket lined with M119, T121, L172, M175, W210ʹ, F217ʹ, F235ʹ, L239ʹ, T243,ʹ and I246ʹ, and anchored within it *via* interactions to D168 with each indole nitrogen (Fig. 3*C*). Lastly, the guanidine group of R242 hydrogen bonds to the maleimide carbonyl.

In structures solved from RedE crystals formed in a solution with only 1.2 mM AFA, we observe electron density consistent with AFA binding the cleft adjoined to the hydrophobic active site pocket (Fig. S3*C*). This secondary binding cleft forms between *β*5, *α*6, and *α*8 of the Rossmann domain and *α*ʹ10 from the C-terminal helical bundle. AFA forms hydrogen bonds from each indole N-H to T169 and a water molecule held between the S165 carbonyl and the D168 sidechain (Fig. 3*B*). In one protomer, H249 may form a weak hydrogen bond to the nearby maleimide carbonyl (3.5 Å). AFA makes hydrophobic contacts with G94, S95, P96, I246, A250, and L253, and the G94 peptide carbonyl is held in place with a hydrogen bond to the S165 side chain. An iodide ion bounded by the positive dipole N-terminal end of *α*4 and R254 completes the outer boundary of the cleft. Despite not including NADP^+^ in the preparation of these crystals, electron density is consistent with the presence of NADP(H) cofactor retained during purification. The electron density has poorer definition than that of the RedE-NADP^+^ or RedE-NADP^+^-AFA structures and can be modeled by partial occupancy of an NADP^+^ cofactor (Fig. S3*A*). Additionally, there is sparse electron density above the nicotinamide moiety resembling partial occupancy of AFA in the same pose as characterized in the RedE-NADP^+^-AFA ternary structure (Fig. S3*B*). The density is a poor fit, and modeling either AFA or water in this area produces similar R_free_ values. Furthermore, placing an AFA molecule in the active site clashes with the clearly defined position of the AFA molecule in the secondary binding cleft. Placing both indolocarbazoles in the active site simultaneously forces a contact between indole rings of less than 2 Å, which is too small to represent simultaneous binding. Modeling this relation as alternate conformations results in occupancy of 55 to 60% in the distal cleft. This feature may result from heterogeneity in binding configurations throughout the crystal.

### Resemblance to other NAD(P)H-dependent reductases

Secondary structure searching *via* PDBeFOLD (34) shows RedE’s closest structural matches are other IREDs and RedAms (Fig. S4). These are defined by the reciprocal domain swapping of each monomer’s C-terminal helical bundle. However, compared to all other IREDs and RedAms RedE has a pronounced crescent shape imparted by the secondary indolocarbazole binding cleft formed between *α*ʹ10 of the dimerization domain and *α*6 of the Rossmann domain. In other IREDs this is a tighter binding interface between protomers, yet buried surface area calculations indicate similar contact areas between 3900 and 4600 Å^2^ (4020 Å^2^ in RedE). The loss of close contacts between *α*ʹ10 and *α*6 in RedE are supplemented with dimer interactions found at the loop between *α*8 and *α*9 set against the N-terminal ends of *α*ʹ6 and *α*ʹ8 of its dimer partner in addition to closer interactions between the *β*-sheet in the Rossmann domain and *α*ʹ9 in the dimerization domain. This extra surface area may provide support to maintain the separation between the domains.

The next closest structural matches are from the *β*-hydroxy acid dehydrogenase (*β*HAD) family, which include 3-hydroxyisobutyrate dehydrogenase (35), l-serine dehydrogenase (36) among several other characterized enzymes (Fig. S4) (37, 38). These share an N-terminal Rossmann NAD(P)H binding domain and a C-terminal helical bundle mediating dimerization; however, a sharp turn in the helix following the interdomain helix (*α*9 in RedE) redirects the chain back toward the Rossmann domain resulting in no reciprocal domain swapping (35). Despite this large deviation, the backbone traces of the overall quaternary structure of the dimer is maintained between IREDs and *β*HADs. The related 6-phosphogluconate dehydrogenases shares a similar fold to *β*HADs, where the helical bundle of a 6-phosphogluconate dehydrogenases protomer has a circa 130 amino acid C-terminal extension which replicates the fold of an interacting *β*HAD dimer (39).

RedE has sparing, but notable, structural similarity with pyrroline-5-carboxylate reductases (40) and prephenate dehydrogenases (Fig. S4) (41). These enzymes share a similar layout to IREDs and *β*HADs with an N-terminal Rossmann binding domain and C-terminal helical bundle mediating dimerization. They even share a similar presence or lack of reciprocal domain swapping resulting from a sharp turn in an *α*-helix in the dimerization domain. However, the organization of the helical dimerization bundle is substantially different with several helices following the long axis of the enzyme rather than being arranged perpendicular to it.

### Domain contacts among IRED and RedAm enzymes

**Figure 4:**
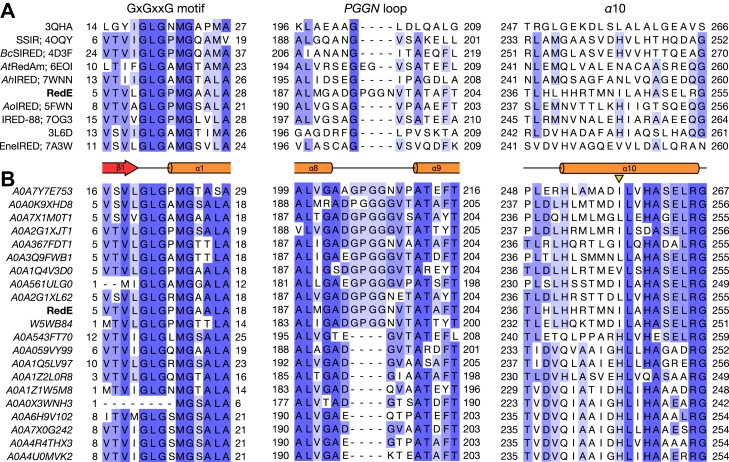
**Multiple sequence alignments of RedE homologues featuring the conserved Rossmann domain nucleotide binding motif (GxGxxG, *left*) the PGGN loop unique to the RedE structure (center) and residues of the *α*10 helix (*right*).***A*, nine of sixteen unique IRED or RedAm hits from secondary structure matching search of the RedE protomer. Sequences are labeled with their name, if applicable, and PDB ID. *B*, the *top* 70 sequence similarity BLAST search hits of the UniProt database find only 10 other IRED-like proteins with a PGGN-like loop, and each retains a hydrophobic residue at position I246 rather than the common histidine found in other IRED-like sequences (*arrow*). After the *top* ten sequences only every fifth sequence is shown in the alignment. *Ao*IRED (PDB: 5FWN) is the 69th search hit. Sequences are labeled with their UniProt ID. IRED, imine reductase; PDB, Protein Data Bank.

Of the 16 unique IRED and RedAm hits from secondary structure matching using the RedE protomer, the median RMSD of C_***α***_ atoms is 2.76 Å covering 93% of residues in RedE (Table S1). Among these enzymes RedE is the only one to have an extended loop between *α*8 and *α*9 which interacts with the Rossmann domain of its partner monomer (Fig. 4*A*). Searching the Protein Data Bank (PDB) for all IREDs and RedAms shows that this is an unique feature to RedE and is an apparent insertion of four residues (194-PGGN-197). *At*RedAm has a two residue insertion in this loop; however, the area does not interact with the neighbouring Rossmann domain (42). Extending the search to primary sequence matches in the UniProt database shows that this “PGGN” loop is found in ten other IRED-like enzymes, though the insertion may be more accurately described as residues 193-GPGG-196 (Fig. 4*B*). This loop interacts closely with P96, V97, D160, A162, and M163 in the Rossmann domain where P96 and D160 are conserved positions among structurally characterized IREDs (Fig. S5). Among more closely related sequences to RedE, the V97 position is found as a hydrophobic residue when the “GPGG” insertion is present and as a glutamate when it is not. AlphaFold predicts these loops adopt a similar conformation to RedE and each predicted assembly possess a cleft between *α*6 and *α*ʹ10 (43).

The sequence alignment of helix *α*10 indicates no residues are strongly conserved among all IREDs, while the loop spanning *β*5 and *α*6 is often conserved as [ST][ST] x [ST]P (Fig. S5). Among structurally characterized IREDs and RedAms a histidine or asparagine is often found at I246 in RedE and is involved in cross-domain contacts (Fig. 4*B*). This position is exclusively found as a hydrophobic residue in other IRED-like sequences with a “GPGG” insertion between *α*8 and *α*9.

### Detection of new StaP indolocarbazole products

Previous characterization of StaP reaction products assayed mixtures after 3 h (22), and given the known propensity for these compounds to decompose quickly, we surmised we may find new compounds produced closer to the start of the reaction. We used an one-pot reaction where the RedE substrate is created *in situ* from CPA by the indolocarbazole synthase StaP with *Synechococcus elongatus* electron transport coenzymes ferredoxin (FDX) and ferredoxin reductase (FDR) (22, 44). Production of reductasporine and StaP products, K252c (**5**, staurosporine aglycone), 7-hydroxy-K252c (**6**), and AFA are verified *via* high-resolution mass and UV-visual absorbance spectra (Table S2). Each indolocarbazole has a diagnostic UV-visual absorbance spectrum with ***λ***_max_ ≈ 290 to 300 nm (13, 45, 46). After quenching these reactions with acetonitrile at 0, 4, 8, 15, 30, and 160 min we see the accumulation and depletion of a new compound with mass consistent with **3** ([M + H]^+^ = 296.11825 m/z, 0.10 ppm), the proposed RedE substrate (Fig. S6*A*). Supplementing reactions with 1 mg/ml catalase in an effort to reduce nonspecific oxidation due to hydrogen peroxide released during incomplete turnover of the StaP cofactor system does not alter the compound’s accumulation or lifespan (Fig. S6*B*). This new compound is accompanied by the detection of two additional, low abundance indolocarbazole compounds with molecular formulas attributable to 6,7-didehydro-K252c ([M + H]^+^ = 310.09755 m/z, 0.19 ppm) and 7-carboxy-6,7-didehydro-K252c ([M + H]^+^ = 354.08724 m/z, 0.25 ppm) (Fig. S7). Including active RedE and RedM under these reaction conditions shows the additional accumulation of a compound consistent with methyl-**3** ([M + H]^+^ = 310.13397 m/z, 0.32 ppm). Mixtures without active RedE do not accumulate this compound, though accumulation of **3** is increased (Fig. S7).

### Single residue mutations in RedE

**Figure 5:**
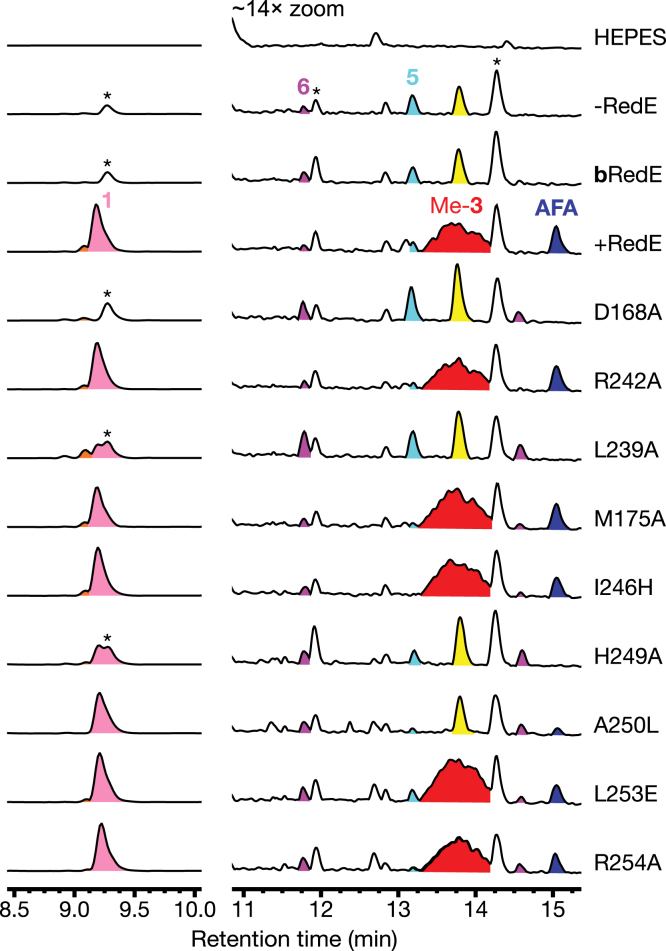
**LC-MS traces of one potreactions of 500 *μ*M CPA, 1 mM NADPH, 1 *μ*M StaP, 5 *μ*M FDR, 20 *μ*M FDX, 20 *μ*M RedM and 20 *μ*M RedE variant held at pH 8 with 20 mM Hepes.** The removal of aspartate from the primary active site *via* D168A eliminates any reductasporine (**1**) production and shifts its metabolite profile to look more like inactivated RedE. L239A in the primary site and H249A in the secondary site both reduce reductasporine accumulation and shifts the metabolite profile to appear like boiled RedE (**b**RedE). A250L does not greatly affect **1** production, but no longer accrues methyl-**3** eluting at 13.8 min. Peaks marked with an *asterisk* (∗) are associated with StaP activity and have no UV-visual absorbance above 230 nm, indicating they are not indolocarbazoles. Their masses correspond to 326, 343, and 371 m/z, from *left* to *right*. The 326 m/z compound adds tailing and shoulders to **1** peaks. Peaks are assigned per UV-visual spectra and mass: *cyan*, K252c (**5**); *purple*, 7-hydroxy-K252c (**6**); *dark blue*, arcyriaflavin A (AFA); *red*, methyl-3; *pink*, reductasporine (**1**). Indolocarbazole peaks with masses 310 m/z (9.2 min) and 342 m/z (13.8 min) are colored *orange* and *yellow*, respectively. CPA, chromopyrrolic acid; FDR, ferredoxin reductase; FDX, ferredoxin.

Based on the ternary cocrystal structures of RedE and sequence alignments, nine single residue variants were generated to investigate the potential role of each binding site for catalytic activity in tandem reactions including StaP, *S. elongatus* FDX, FDR and RedM (Fig. 5). RedM is included to methylate the RedE product (**2**), as it has a short lifespan in solution. In the primary site, the D168A variant targets the position often found to be sensitive to mutation in many IREDs and *β*HADs (31). The role of the residue at this position is not fully understood, and mutation can eliminate or reduce catalytic activity or alter stereoselectivity. The D168A variant does not accumulate methyl-**3** or reductasporine, suggesting a critical role for this residue. Next, we sought to make mutants that would perturb the hydrophobic lining of the primary site. L239 presses directly on the maleimide ring of AFA forcing it to pack closely to the nicotinamide ring of the cofactor, while the more distant M175 packs with L172, Wʹ210, Fʹ217, and Fʹ235 and together form half of the active site pocket. Assays of the L239A variant result in reduced reductasporine formation, whereas M175A has activity similar to the WT RedE. Lastly, residue R242 hovers above the active site near the maleimide ring of AFA and may participate in substrate recognition. It is also a rare large residue to find in this position (Fig. 4*B*) and may act to prevent a close fit between domains. However, the R242A variant has identical activity to WT RedE.

In the secondary site, H249 is positioned to hydrogen bond with indolocarbazole substrates and may stabilize **3** *via* its pyrrolic nitrogen or may interact with a carboxy group of a carboxylated precursor (such as **4**). The H249A variant has markedly reduced reductasporine accumulation, and no methyl-**3** is observed. The A250L variant has normal reductasporine production; however, the production of methyl-**3** is eliminated. Observing these variants on short time scales in the absence of RedM shows the pronounced accumulation of [M + H]^+^ ions attributable to 6,7-didehydro-K252c and 7-carboxy-6,7-didehydro-K252c (Fig. S8). Together these indicate that the secondary binding site plays a role in the flux of indolocarbazole compounds through the biosynthetic pathway. Sequence analysis of IREDs found in the PDB and enzymes most similar to RedE suggest there are several residues involved in domain-domain contacts that are not preserved in RedE. For example, IREDs will often have a glutamate or aspartate in place of L253 in RedE and a histidine or asparagine in place of I246, which interact with residues of the nearby *α*6 helix and loop of the Rossmann domain. However, replacing these residues in RedE, as in variants L253E and I246H, results in no change from WT activity. Lastly, R254 forms the back wall of the secondary binding cleft and interacts with the unique PGGN loop. We hypothesized that changes to this residue could destabilize the loop and widen the secondary site, but RedE-R254A mutants behave similarly to the WT enzyme.

## Discussion

Structures of RedE bound with the substrate mimic AFA unambiguously show two indolocarbazole binding sites. The primary binding site positions the C5 atom immediately above the nicotinamide ring of the NADPH cofactor for hydride transfer, and the secondary binding site places the indolocarbazole in an adjoining cleft formed between the dimerization domain and Rossmann domain. No secondary binding site has been described among other IREDs and RedAms; however, a *Pseudomonas putida* putative oxidoreductase with structure coordinates deposited in the PDB (PDB: 3L6D, 24% identity) features a similar cleft separating these domains. Given that this unique structural feature is important to catalysis in RedE as determined by mutations, we sought out structural and sequence features distinguishing RedE from other IREDs and RedAms which may be involved in the creation of this cleft.

With the increased publication of IRED structures over the past ten years, it has become well-established that the Rossmann domain rotates relative to the dimerization domain into distinct open and closed conformations with at least seven IRED and RedAm enzymes captured in either state (42, 47, 48, 49, 50, 51, 52). These domain movements are often seen when comparing apo structures to binary or ternary complexes. Superposing open and closed conformation protomers onto RedE shows that RedE resembles an exaggerated open conformation, with all atom RMSD values on average 0.3 Å lower for open conformations as to closed conformations. RedE shows no conformational changes upon cofactor or ligand binding, aside from the reorganization of the loop after *β*2 upon NADP^+^ binding positioning R33 to interact with the adenine ring and 2ʹ-phosphate of the cofactor. Given the ternary complexes of RedE have little to no room for the Rossmann domain to move toward the dimerization domain due to the tightly packed AFA in each binding site, the structure of RedE may represent a maximally closed conformation despite resemblance to other IRED open conformations. The open to closed transition in these other IREDs hinge at the N-terminal end of the interdomain helix (RedE: *α*8) just below the contact point between *α*6 and *α*ʹ10. A prominent domain-domain bridging interaction is often found here as a glutamate residue at position 250 or 253 (RedE numbering) whose side chain associates with the N-terminal dipole moment of *α*6 while hydrogen bonding (8, 10, 47, 48, 49, 50, 53, 54, 55, 56, 57), or as a salt bridge between an arginine from *α*ʹ8 to an aspartate or glutamate on helix *α*6 (51, 52). RedE does not preserve either contact point, instead P194 and G195 from the “PGGN” insertion loop connecting *α*ʹ8 to *α*ʹ9 (Fig. 4) extends out to residues P96, V97, and R100 of *α*6, and D160, A162, M163, and V166 of the N-terminal of *α*8. This is the only interdomain contact to *α*6 aside from Rʹ254 in *α*ʹ10 *via* an iodide ion associated with the helix’s N-terminal positive dipole moment. Removing this interaction in the R254A variant leaves RedE with WT activity levels and likely does not play a significant role in stabilizing the enzyme’s structure. The second series of contacts between domains of IRED and RedAm enzymes occur between *α*ʹ9 and *β*6-8, which are largely hydrophobic. Many enzymes have a series of large and medium sized residues arranged at this interface, such as tryptophan, phenylalanine, tyrosine, and histidine (8, 9, 47, 48, 49, 51, 52, 53, 55, 57). Other IRED and RedAm enzymes which do not have large residues at these sites do not have intimate interdomain contacts between *α*ʹ9 and the *β*-sheet of the Rossmann domain, and the majority of domain-domain interactions occur at *α*ʹ10 (10, 42, 50, 56, 58, 59). RedE has a series of small residues in *α*ʹ9 (A202, V206, and T213) which interlock with *β*-sheet residues (L131, L133, M155, and L157), resulting in a tight interdomain interface. The combination of loose interactions between *α*ʹ10 and *α*6 alongside a tight interface between *α*ʹ9 and *β*6-8 results in the Rossmann domain being pulled down toward *α*ʹ9 and away from *α*ʹ10 relative to other IREDs, creating the exaggerated open conformation and secondary indolocarbazole binding cleft.

The IRED-like enzyme from *P. putida* KT2440 (PDB: 3L6D) has a similar interdomain cleft between helices *α*ʹ10 and *α*6. We find a similar domain-domain contact pattern as RedE with loose to no direct connections between *α*ʹ10 and *α*6, and a tight interface between *α*ʹ9 and *β*6-8 with mainly small interlocking residues. Notably, the N-terminal of the interdomain helix tucks W169 underneath *α*6, imparting a large rotation to the Rossmann domain away from the interdomain helix relative to RedE. Also, *α*ʹ9 adopts a kink not seen in other IRED structures between residues 214 to 218 allowing the relatively large F217 to pack parallel to the Rossmann domain B-sheet rather than push against it.

In IRED and RedAm enzymes an active site aspartate or tyrosine is typically found to be essential for catalysis (31). *β*HAD enzymes are found to use lysine residues at this position in order to catalyze ketone reductions. RedE maintains this active site aspartate (D168); however, it is solely used to anchor the indolocarbazole substrate in the primary active site *via* hydrogen bonding to the two indole N-H moieties. Inspection of the active site reveals there are no candidates for a catalytic base in RedE, with the only protic residue within a 5 Å sphere of the AFA maleimide group being R242, an unlikely proton donor. Furthermore, the R242A mutant has no impact on enzymatic reaction products. Additionally, StaP is capable of producing an unstable product consistent with **3**, the proposed isoindole substrate for RedE. Isoindole readily tautomerizes in solution despite its 10*π* electron aromatic ring system (60). The planar, aromatic 2H-isoindole tautomer dominates in polar aprotic solvents, whereas the imine 1H-isoindole tautomer dominates in nonpolar or protic solvents (60, 61). Therefore, if the 26*π* electron system of **3** acts analogously to isoindole, the imine form should dominate in the primary active site either due to water exposed isoindole nitrogen or preequilibration to the imine form from solution or secondary binding site. Without a dedicated catalytic base and with the reactive imine form of the substrate potentially favored in solution the subsequent reduction *via* NADPH appears to be catalyzed in RedE by simple substrate orientation and desolvation effects. When the close packing of the primary site around the isoindole nitrogen is disturbed, as in L239A, the overall production of products is greatly reduced. This may be due to the increased mobility of the indolocarbazole and consequent loss in precision alignment of the substrate for efficient hydride transfer.

The unexpected distal secondary indolocarbazole binding site formed at the gap between the C-terminal helical dimerization domain and N-terminal Rossmann domain implies a mechanism for unstable substrate management. Compound **3** is found to be unstable, mostly disappearing after 2 h *in vitro* (Fig. S6). This is consistent with other isoindole compounds that are known to oxidize readily when exposed to aerated solutions (61) and form compounds analogous to known StaP oxidation products (20). The secondary binding site may sequester indolocarbazole substrate from solution, protecting it from autooxidation and enabling subsequent reduction. Disruption of the secondary site asserts that the site is essential to efficient catalysis with mutants H249A and A250L resulting in losses in activity and accumulation of off-pathway indolocarbazole products. By the structure, A250L sterically excludes indolocarbazoles from binding, whereas the effect of the H249A mutation is less trivial to explain. H249 could play a role in substrate recognition where it would hydrogen bond to the isoindole nitrogen locking the substrate into place within the secondary site. This hydrogen bond may act as a pseudo N-substitution lessening the nucleophilic character at C5 and C7 as seen in the more stable N-substituted isoindole molecules, which tend to favor the planar, aromatic tautomer (60, 61). Alternatively, H249 may play an active role in selecting for the particular imine tautomer that lines up with the NADPH cofactor for reduction (Fig. S9). This implies a direct transfer of the imine substrate from the secondary site to the primary site. Such a transfer could be guided by D168, as an indole nitrogen is available for hydrogen bonding from the secondary site to D168 if the side chain rotates and displaces the nearby water molecule (Fig. 3*B*). With this hydrogen bond in place the side chain can swing back down and guide the indolocarbazole from the secondary site to the primary site. These ideas of substrate protection and tautomer selection determining the flux of indolocarbazole products down a productive biosynthetic route mirrors the proposed mechanisms distinguishing RebC and StaC activity in the generation of the aglycone cores of rebeccamycin and staurosporine, respectively (22, 23, 25). RebC and the StaC-like RebC mutant, RebC-10x, were each crystallized with 7-carboxy-K252c in their active sites, but in alternate tautomeric forms (23, 25). RebC traps the enol form requiring decarboxylation-oxidation in order to form AFA (23), whereas RebC-10x traps the keto form, making the carboxylate group available for decarboxylation directly to K252c, the staurosporine aglycone (Fig. S1*B*) (25). The secondary active site we report here for RedE represents a novel approach to the similar issue of handling short-lived substrates in indolocarbazole biosynthesis.

Over the past ten years IREDs and RedAms have drawn increased attention over their potential application to the synthesis of chiral amine products for use in drug manufacturing (3, 4, 5, 6). RedAm substrate scopes have previously been limited to combinations of small ketone/amine molecules, with recent efforts directed at identifying and engineering enzymes capable of accommodating larger molecules (50, 51, 57, 62, 63). Tantalizingly, the RedE structure shows that the combined indolocarbazole binding cleft is some 24 Å wide providing ample space for engineering efforts toward effecting arbitrary reductive aminations. Alternatively, RedE may be used to identify the sequence determinants of an extended binding cleft in order to create them in proven RedAms for expanded substrate scope.

In summary, we report the structure of the reductasporine biosynthetic IRED RedE cocrystallized with AFA revealing an unprecedented extended active site cleft capable of binding two indolocarbazole molecules. The structure of and site-directed mutagenesis in the primary binding site suggests reduction is catalyzed *via* precise substrate orientation and desolvation effects, and the secondary binding site may serve to capture and protect indolocarbazole substrates from spontaneous oxidation. This secondary site may even select for the imine tautomer readily reduced in the primary site when transferring directly from secondary to primary active site. Approaching IREDs from their context in biosynthesis can serve us unique insight into the family *via* unique structural variations and natural substrate scope, and RedE provides a promising new protein scaffold for reference in iterative engineering.

## Experimental procedures

### General methods

Primers were synthesized by Integrated DNA Technologies. Q5 DNA polymerase, T4 DNA ligase, and restriction endonucleases were purchased from New England Biolabs. DNA sequencing was carried out by either Nucleic Acids Protein Service Unit DNA Sequencing Facility or CMMT/BCCHR DNA Sequencing Core Facility (University the British Columbia). Nickel sepharose protein purification resin and HiLoad 26/60 Superdex 200pg size exclusion column was purchased from GE HealthCare. Other general reagents were purchased from Anatrace, Bio Basic Inc, Hampton Research, Sigma-Aldrich, Thermo Fisher Scientific, and VWR International as necessary. Liquid chromatography was carried out on either an Agilent 1260 Infinity or Infinity II HPLC system with diode array. Low-resolution mass spectrometry data were recorded on an Agilent 6120 Quadruple LC/MS, and high-resolution LC-MS data was recorded on an Agilent 6546 LC/quadrupole time-of-flight machine. Indolocarbazoles were separated on C18 columns sourced from Phenomenex and Agilent (250 × 4.6 mm, 5 *μ*m particle size).

### Cloning, expression, and purification of redE and redM

Genes *redE* and *redM*, codon optimized for expression in *E. coli*, were cloned into pET28a at the NdeI and XhoI restriction sites with a stop codon was included before the XhoI cut site, leaving an N-terminal 6xHis tag in the gene product. Plasmids pET28a-*redE* or pET28a-*redM* were transformed into calcium-competent *E. coli* BL21(DE3) by heat-shock and grown in lysogen broth (LB; 10 g/L NaCl, 10 g/L tryptone, 5 g/L yeast extract) (37 °C, 180 rpm) with kanamycin (50 *μ*g/ml) to an *A*_600_ of 0.6 before cooling to 16 °C. Expression was induced by addition of IPTG (to 100 *μ*M) and incubated at 16 °C for a further 18 h. Cells were pelleted by centrifugation (4 °C, 10 min, 3000*g*), and resuspended in 50 mM Tris (pH 8), 500 mM NaCl, 5 mM imidazole supplemented with either 1.5 mM tris(2-carboxyethyl)phosphine (TCEP) or 5 mM 5 mM β-mercaptoethanol (BME) reducing agent. Cell suspensions were lysed by sonication (25% amplitude, 4 s on/8 s off cycles, 6 min), and cell debris removed *via* centrifugation (45 min, 15,000*g*). The supernatant was loaded onto Ni-IDA affinity resin and eluted with a 20 to 500 mM imidazole step gradient in 50 mM Tris (pH 8), 500 mM NaCl, and either 1.5 mM TCEP or 5 mM BME. Elution fractions with recombinant protein were passed through a 0.22 *μ*m syringe filter and purified *via* size-exclusion chromatography (20 mM Tris (pH 8), 50 mM NaCl with corresponding reducing agent. SDS-PAGE was used to track the purity of resulting fractions for further use in crystallography screens.

### Selenomethionine incorporation into RedE

*E. coli* BL21(DE3) transformed with pET28a-*redE* were grown in LB medium with kanamycin (50 *μ*g/ml) overnight. From this culture, a 30 *μ*l aliquot was inoculated into 30 ml of antibiotic supplemented M9 medium for growth overnight. Starter cultures (10 ml) were each added to 1 L of M9 medium with kanamycin and grown at 37 °C (180 rpm) for 4 h to an *A*_600_ of 0.7 before cooling to 16 °C over 30 min. Lysine, threonine, and phenylalanine (100 mg each) followed by leucine, isoleucine, valine, and selenomethionine (50 mg each) were added to 1 L of medium (64). Cultures were then induced with 100 *μ*M IPTG and grown for 18 h. Selenomethionine-substituted RedE was purified as described previously with buffers supplemented with 5 mM BME.

### RedE crystallization

Purified protein was screened on a variety of commercially available screens (Hampton, Index, Top96, MCSG) in a 96-well sitting drop format with 2 *μ*l drops (1:1 ratio of protein to precipitant solution). Preliminary hits were optimized in a hanging-drop format with drops mixed in a 1:1 ratio of well to protein solution. Diffraction quality RedE crystals were grown over wells containing 0.33 M NaI, 0.1 M Tris pH 8.5, 21 to 23% PEG 3350 and 1.5 mM TCEP or 5 mM BME using up to 42 mg ml^−1^ of protein. Under these conditions RedE cocrystallized with NADP^+^ (100 mM in water) and/or AFA (100 mM in dimethyl sulfoxide) when 10 mM and 1.2 mM of each, respectively, were included in the protein solution. Crystals of apo-RedE grew at 0.35 M NaI, 0.1 M bicine pH 9, 25% PEG 3350 and 5 mM BME. Selenomethionine-substituted RedE crystals were prepared similarly with mother liquor at 0.3 M NaI, 0.1 M Tris pH 8, 25% PEG 3350 and 10 mg ml^−1^ protein. All crystals were soaked for 1 to 10 min in mother liquor prepared to 10 to 15% ethylene glycol before flash-freezing in liquid nitrogen. Crystals were tested for diffraction using a Rigaku MicroMax-007HF (1.54 A, rotating copper anode) with Saturn 944+ charge-coupled device detector, and those diffracting to at least 3 Å were retained for data collection at a synchrotron.

### Data collection, structure determination, and refinement

Datasets for the selenomethionine-substituted RedE were collected on beamline 08ID-1 at the Canadian Light Source using MX300-HE and Pilatus 6 M detectors. Datasets for RedE cocrystals were collected on beamline BL9-2 at the Stanford Synchrotron Radiation Lightsource. Data sets were integrated using iMOSFLM (https://www.mrc-lmb.cam.ac.uk/mosflm/mosflm/) (65) or XDS (https://xds.mr.mpg.de/) (66) and merged in AIMLESS (67). Selenomethionine structures were phased *via* single anomalous dispersion of selenium and iodine atoms *via* the Autosol tool in the Phenix (68) software package (https://phenix-online.org/). The single-wavelength anomalous diffraction phasing results were then input into Autobuild (69) for initial model building followed by several rounds of manual inspection and building in COOT (https://www2.mrc-lmb.cam.ac.uk/personal/pemsley/coot/) (70) and refinement *via* phenix.refine (71). Native protein datasets were processed similarly with phases solved for *via* molecular replacement using the SeMet-RedE model through PHASER-MR (https://www.phaser.cimr.cam.ac.uk/) (72). Ligand restraints for AFA were generated using eLBOW (73).

### Site-directed mutagenesis of RedE

Primers were designed so that the overlap region containing the mutation had a T_m_ ∼55 °C and the nonoverlapping region had a T_m_ about 7 °C higher (calculated against the complementary DNA strand) (74). Where possible mutations were included that removed or added restriction sites for preliminary screening *via* digests. Standard PCR was carried out using an annealing step set to the higher T_m_ and ended with a 10 min elongation step. The PCR product mixture was incubated with DpnI before transforming the digest solution as described previously into *E. coli* DH5*α*. Purified plasmids were screened for restriction fragment polymorphism and mutations confirmed by sequencing.

### Cloning and expression of rebO and rebD, and production of CPA

Genes *rebO* and *rebD* were amplified from genomic DNA (*Lechevalieria aerocolonigenes* DSM 44217), and ligated into pET28a and pET22b, respectively. These plasmids were transformed into chemically competent *E. coli* BL21 (DE3) cells and used to prepare CPA according to the previously published procedure (17).

### Expression and purification of StaP, FDX, and FDR

Plasmid pET28a-*staP* was kindly provided by Catherine Drennan (Massachusetts Institute of Technology), and plasmids pET28a-*fdr* and pET28a-*fdx* coding for *S. elongatus* PCC 7942 ferredoxin-NADP oxidoreductase (FDR) and FDX I, respectively, were received from Dr Hai-Yan He (Institute of Medicinal Biotechnology, Chinese Academy of Medical Sciences).

*E. coli* BL21(DE3) transformed with pET28a-*staP*, pET28a-*fdr* or pET28a-*fdx* were grown in LB medium with kanamycin (50 *μ*g/ml) to an *A*_600_ of 0.5 before cooling to 16 °C over 30 min. Each culture was then supplemented with 100 *μ*M IPTG and cells with plasmids coding for heme proteins (StaP and FDX) were further supplemented with 1 mM *δ*-aminolevulinic acid before incubating for 18 h.

Media were removed by centrifugation (4 °C, 10 min, 3000*g*), and cells resuspended in 50 mM Tris (pH 8), 500 mM NaCl, and 5 mM imidazole. The bacterial suspension was lysed *via* sonication (25% amplitude, cycles of 4 s on and 8 s off for 6 min), and cell debris cleared *via* centrifugation (45 min, 15,000*g*). Supernatant was passed through a Ni-affinity column and enzyme harvested over an imidazole step gradient (5–300 mM). Fractions containing recombinant enzyme were dialyzed to remove excess imidazole and salt before screening for activity against CPA followed by adding glycerol to 10% (v/v) and storing at −70 °C.

### Analysis of RedE variants

A typical reaction includes 500 *μ*M CPA incubated with 1 *μ*M StaP, 20 *μ*M RedM, and 20 *μ*M RedE variant in the presence of 1 mM NADPH, 5 *μ*M FDR and 20 *μ*M FDX cofactors held at pH 8 with 20 mM Hepes. Reactions are quenched with the addition of an equal volume of acetonitrile (ACN) and analyzed on a reverse phase C18 HPLC column (1 ml min^−1^, 98% H_2_O to 98% ACN gradient over 30 min, each solvent supplemented with 0.1% formic acid). Elution was monitored using a diode array from 220 to 500 nm or by mass.

### Time-course analysis of StaP reaction products

StaP reactions include 500 ***μ***M CPA, 1 mM NADPH, 1 ***μ***M StaP, 5 ***μ***M FDR, and 20 ***μ***M FDX held at pH 8 with 20 mM Hepes at room temperature. Reactions were quenched with equal volumes of ACN and analyzed on a reverse phase C18 HPLC column (1 ml min^−1^, 98% H_2_O to 98% ACN gradient over 18 min, each solvent supplemented with 0.1% formic acid). Elution was monitored at 280 nm and by mass.

### Visualization and analysis software

Pymol was used for secondary structure matching and creating protein visualizations (75). Surface areas and dimer interaction surfaces were calculated *via* PISA (33). Protein sequence alignments were generated using Clustal Omega (34) or COBALT (https://www.ncbi.nlm.nih.gov/tools/cobalt/) (76) and visualized with Jalview (https://www.jalview.org/) (77).

## Data availability

Crystal structure model coordinates and diffraction data for apo-RedE, RedE bound with NADP^+^, and complexes of RedE with NADP^+^ and arcyriaflavin A in either primary or secondary site can be found at the Protein Data Bank under accession codes 8U04, 8U05, 8U06, and 8U07, respectively.

## Supporting information

This article contains supporting information.

## Conflict of interest

The authors declare that they have no conflicts of interest with the contents of this article.
