# Supplementary material for: An imine reductase that captures reactive intermediates in the biosynthesis of the indolocarbazole reductasporine
Source: J Biol Chem. 2024 Jan 8;300(2):105642. doi: 10.1016/j.jbc.2024.105642 (PMC10851217; doi:10.1016/j.jbc.2024.105642)
Supplement: Supporting Information [file mmc1.pdf]

Supporting Information for

An imine reductase that captures reactive intermediates in the biosynthesis of the indolocarbazole reductasporine

Phillip Daniel-Ivad[1] and Katherine S. Ryan[1,*]
[1] Department of Chemistry, The University of British Columbia, Vancouver, British Columbia, Canada
*For correspondence: Katherine S. Ryan, Email: ksryan@chem.ubc.ca

Codon-optimized nucleotide sequences and primers for plasmid construction *1*
Site-directed mutagenesis primers *2*
Figure S1: Indolocarbazole biosynthesis; common steps and unique tailoring *3*
Figure S2: Selenomethionine-substituted RedE model *4*
Figure S3: Ligand electron density mF_o–DF_c omit maps *5*
Figure S4: Comparison of the IRED fold to structurally similar dehydrogenase families *6*
Figure S5: Sequence alignments of PGGN loop interacting residues and β5-β6 loop *7*
Figure S6: Time course of StaP reaction products *8*
Figure S7: Accumulation of RedE substrate **3** and methyl-**3** *9*
Figure S8: Time course of StaP and RedE variant reaction products *10*
Figure S9: Potential substrate tautomerization via H249 .... *11*
Table S1: RedE secondary structure matching search results *12*
Table S2: UV-Vis spectra and high-resolution masses of indolocarbazole compounds *13*

RedE plasmid assembly materials:
Amino acid sequence: UniProt ID: A0A0F7G0Y4, GenBank Accession: AKG47111.1

```
>Codon-optimized RedE nucleotide sequence
ATGGGTGCGAAAGTGACCGTTCTGGGTCTGGGCCCGATGGGGGCTGCGTTAGCGGGAGCCTTTTTGGCGGCTGGACA
TCGTACTACGGTGTGGAATCGGACGCCTGGCAAAGGCGGCAGTCTGGCAGGCGAAGGCGCAACTGAGGTGGCTAGTG
CGGCCGAGGCTGTGGCCGCGAGTCCCTTGGTGGTCGTGTGTTTAGCCACGTACGAAGCAGTGCATGAAGTGCTGGAT
CCGCTGGCCGATGAACTGGCCGGTCGTACCGTGGTGAATCTTACGTCCGGGTCGCCGGTGCATGCCCGCGAAACCGC
GAATTGGGCGCAGCAGCATGGTGCGGAATATCTGGATGGCGTGATTATGACCACCCCGAGCGGCATAGGCAAACCGG
ATTATCTGTTGCTGTATAGCGGCTCTCAGGCGGCGTTTGATGGCAGCCGTGGCACCTTATGCGCGCTGGGCGAGCCC
ATGAATTTGGGAACCGATGCAGCGATGGCCTCGGTGTATGATACCGCGCTGCTGGGCCTGATGTGGGGCACCCTGAC
CGGCTGGCTGCACGGCGTAGCGCTTATGGGCGCTGACGGCCCAGGTGGCAACGTCACCGCGACCGCGTTTACCGAAG
TGGCAAATCGTTGGATGAAGACTGTGGGCGTGTTTATGAATACCTATGCGCCGCATGTGGATGCGGGGCATTATCCG
GGGGATGAGTTTACCCTGCATCTGCATCACCGTACCATGAACATTCTTGCGCATGCAAGCGAACTGCGTGGCGTTGT
GAGCGGCCTGCCGGAATTGCTGACCGAACTGACGGGCCGTGCGATTACCGCAGGACATGGCAATGATAGCTACGCTC
GTCTGGTGGAATTCATTCGTAAAGATGGTAGCCCGATT
```

Forward primer: GAACGACAT**ATG**GGTGCGAAAGTGAC (NdeI cut-site underlined, start codon bolded)
Reverse primer: CAACCTCTCGAG**TTA**AATCGGGCTACCATCT (XhoI cut-site underlined, stop codon bolded)

RedM plasmid assembly materials:
Amino acid sequence: UniProt ID: A0A0F7G196, GenBank Accession: AKG47110.1

```
>Codon-optimized RedM nucleotide sequence
ATGAGCGACACCTCTCCAGGTCCTGAGCACGCACCAGCAATCGACCGTCTGCTGCAAATCGCTACCGGTTTCATGGC
CAGCAAGGTGCTGCTGGTGGCAGCATCTCTGGGTCTGTTCACGGAGCTGGCAGCTGGTCCTCTGCGTGGTGAAGAGC
TGCGTGCACGTCTGCGTCTGCATCCACGTTCTGCACGTGATTTCTTCGACACGCTGGTCGCACTGGGTGTCCTGGAG
CGTACTAACGGTGCTTACGCAAACACGCCGGCAACTGCTCAGTACCTGGTGCGTGGTAAATCCGCGTACCTGGGCGG
TCTGCTGGAGATGTCCGACGCTCGTATGTACGAGCTGTGGGGTCGTCTGGACGAGGGTCTGCGTACTGGTAACCCGC
AAAACGAAATCCGCACCGGTGAAGAAGGCATCTACGCCACCCTGTACGATGACCCGGACCGTCTGGACGCTTTCCAG
CAGGCTATGACCGGTCTGAGCATGCGTTCCGCTCACGCTCTGGCTGAAGCTATCGACTGGAGCGCTTACCGTACCGT
GGCGGATATCGGTTGCGCGGAAGGTACCGTTCTGATCCACCTGCTGGAACGTCACCCGCACCTGCGTGGTACTGGCT
TCGATCTGGCCGCGGTTCGTCCGTCTTTTCAGCGTCGCCATGAAGAATCTGGCCTGGGCGATCGCCTGGCGTTTCGC
GCGGGCGATTTTTTTGCGGAACCGCTGCCGCAGGCCGATGCGCTGGTATTCGGCCATATTCTGTCTAATTGGGCGCT
GCCGAAAGCGAAAACTCTGCTGCGCAAAGCCCACGAAGCGCTGCCGGAAGGCGGCATTGTAGTAATCTATGAAACCC
TGATTGACGACGAACGCCGCGAAAACGTTCCGGGCCTGCTGATGTCCCTGACCATGCTGCTGGAAACCCCGGGCGGC
TTCGAATATACCGGCGCCGATTGTCGCGAATGGCTGGCGGATGCGGGCTTCCGCGAATCCCGCGTTCAGTATCTGGC
GGGCCCGGAAAGCATGGTTATTGCCACTAAA
```

Forward primer: CAGCAGCAT**ATG**AGCGACACCTCTC (NdeI cut-site underlined, start codon bolded)
Reverse primer: CGGATTCTCGAG**TTA**TTTAGTGGCAA (XhoI cut-site underlined, stop codon bolded)

Site-directed mutagenesis primers:

Name*	Sequence
RedE-D168A-F	ATGGCCTCGGTGTATGCTACCGCGCTGC
RedE-D168A-R	GCAGCGCGGTAGCATACACCGAGGCCAT
RedE-M175A-F	GCTGCTGGGCCTGGCGTGGGGCACCCTG
RedE-M175A-R	CAGGGTGCCCCACGCCAGGCCCAGCAGC
RedE-L239A-F	CTGCATGCGCATCACCGTACCATGAACATTCTTGCG
RedE-L239A-R	GTGATGCGCATGCAGGGTAAACTCATCCCCCGG
RedE-R242A-FspI-F	CATCACGCTACCATGAACATTCTTGCACATGCAAGC
RedE-R242A-R	CATGGTAGCGTGATGCAGATGCAGGGTAAACTCATCC
RedE-I246H-FspI-F	CCATGAACCATCTTGCACATGCAAGCGAACTGCGTGG
RedE-I246H-FspI-R	GTGCAAGATGGTTCATGGTACGGTGATGCAGATGCAGGGTAAAC
RedE-H249A-FspI-F	TTCTTGCGGCTGCAAGCGAACTGCGTGGCGTTGTGAGC
RedE-H249A-FspI-R	GCTTGCAGCCGCAAGAATGTTCATGGTACGGTGATGCAGATGC
RedE-A250L-FspI-F	CTTGCACATTTAAGCGAACTGCGTGGCGTTGTGAGCG
RedE-A250L-FspI-R	CGCTTAAATGTGCAAGAATGTTCATGGTACGGTGATGCAGATGCAG
RedE-L253E-F	CAAGCGAAGAGCGTGGCGTTGTGAGCGGCCTGCC
RedE-L253E-FspI-R	CCACGCTCTTCGCTTGCATGTGCAAGAATGTTCATGGTACGG
RedE-R254A+XhoI-F	GCGAGCTCGCTGGCGTTGTGAGCGGCCTGCCGGA
RedE-R254A+XhoI-R	CGCCAGCGAGCTCGCTTGCATGCGCAAGAATGTTCATGGTACGG

*FspI and XhoI cut-site removed or added, respectively, as noted.

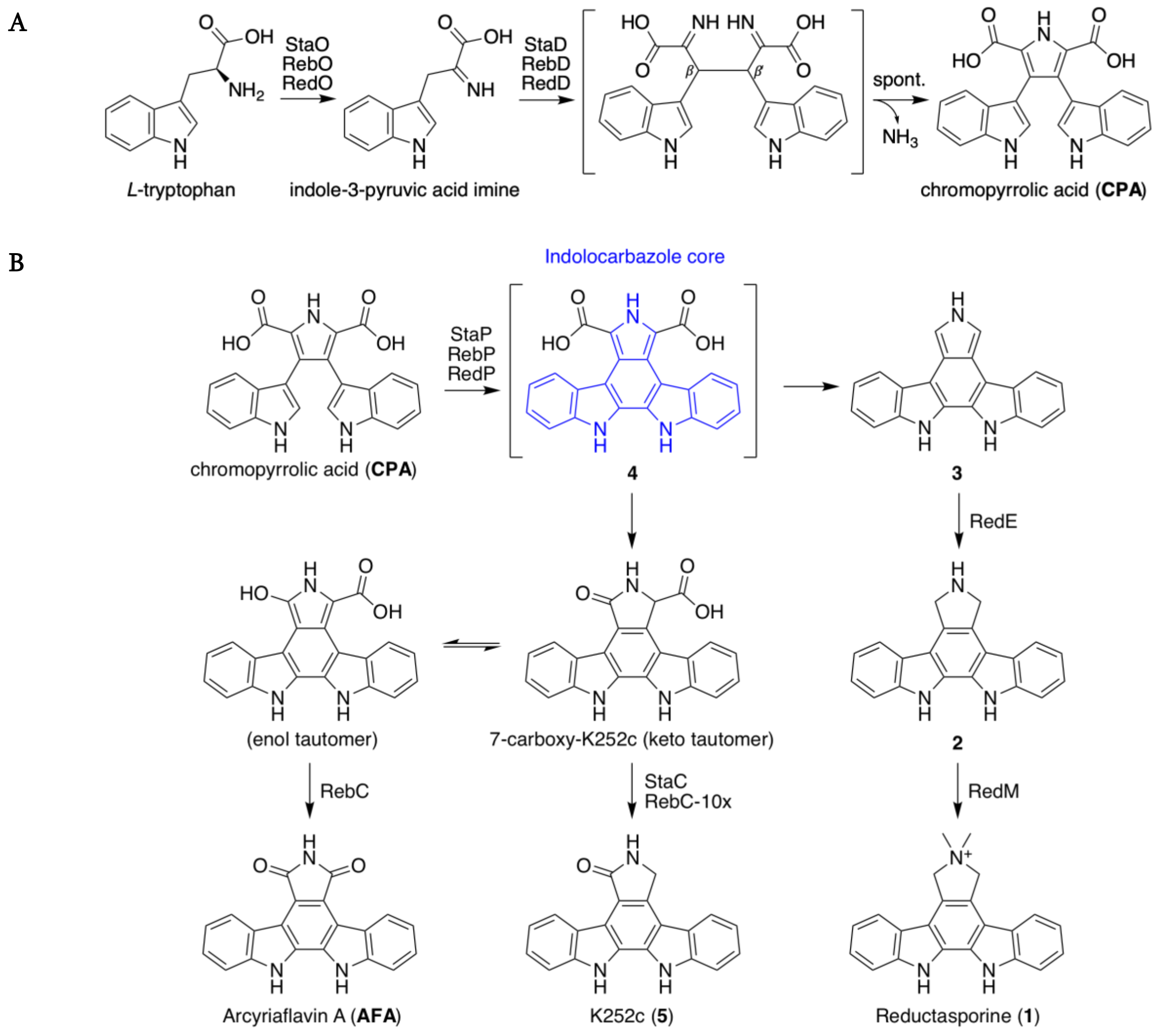

Figure S1. Examples of indolocarbazole biosynthesis. (A) The conserved catalytic steps to produce chromopyrrolic acid. (B) Formation of the indolocarbazole core from CPA and the proposed tailoring steps catalyzed by RebC (rebeccamycin biosynthesis), StaC (staurosporine biosynthesis) and RedE (reductasporine biosynthesis).

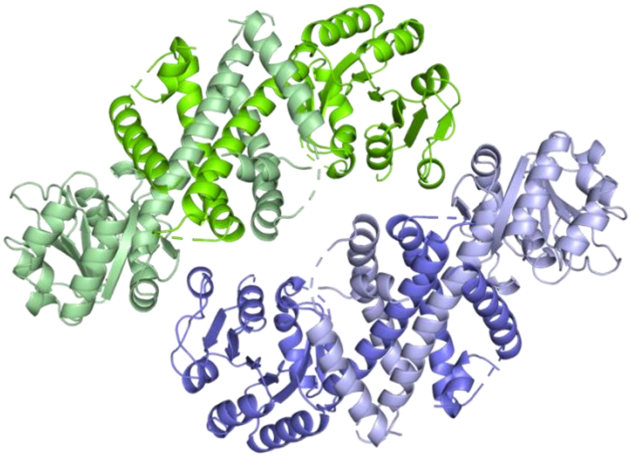

Figure S2. Asymmetric unit of the selenomethionine-substituted RedE structure. The model is missing residues in several loops (A24, G25, K127-Y130, P194-N197, Y230-F235) and α11 (residues 256-278) due to poor electron density.

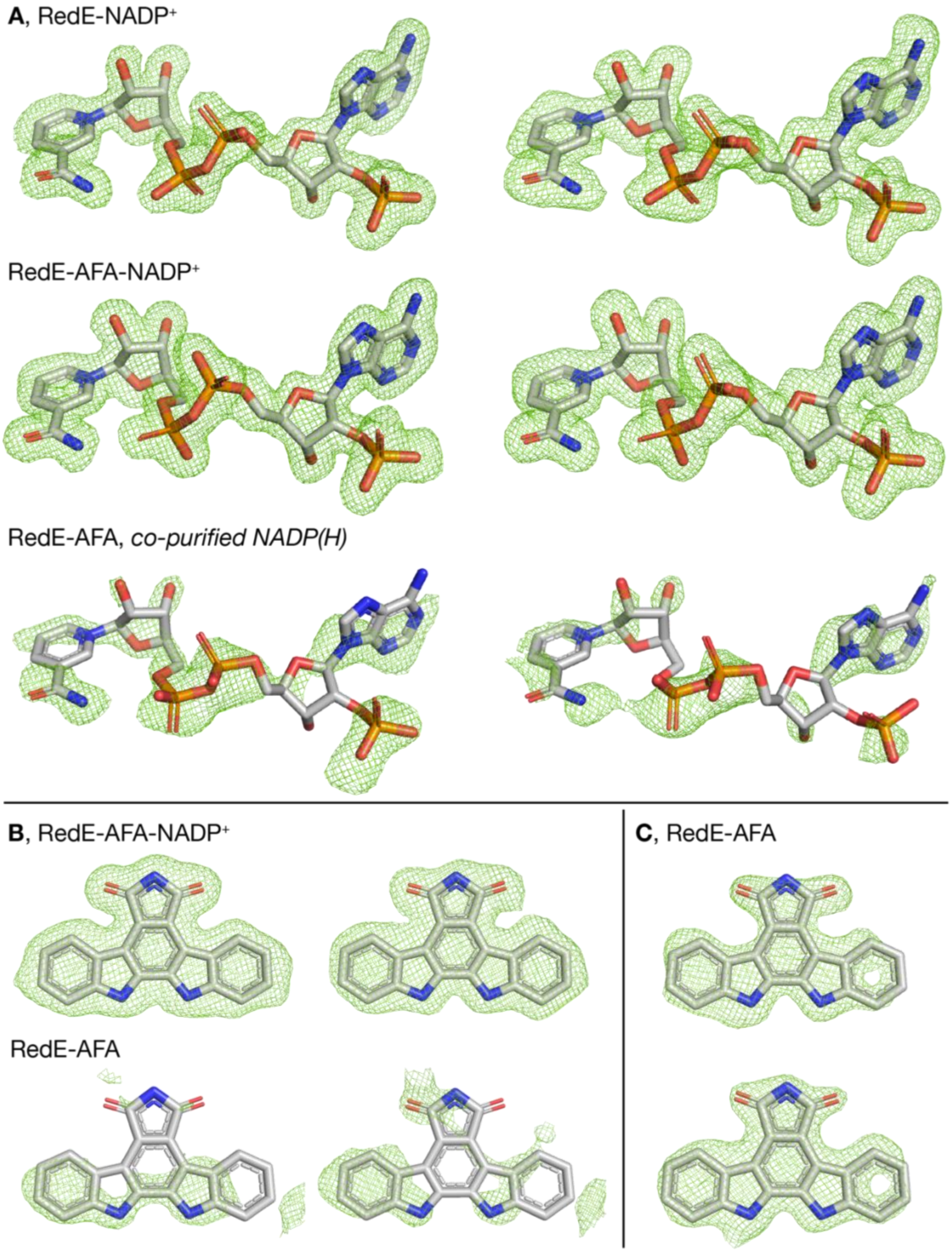

Figure S3. Ligand mF_o-DF_c omit maps contoured at 3σ of (A) NADP+ cofactor, (B) primary site arcyriaflavin A (AFA) and (C) secondary site AFA. NADP(H) cofactor co-purified with the RedE-AFA co-crystal is modelled as $NADP^+$.

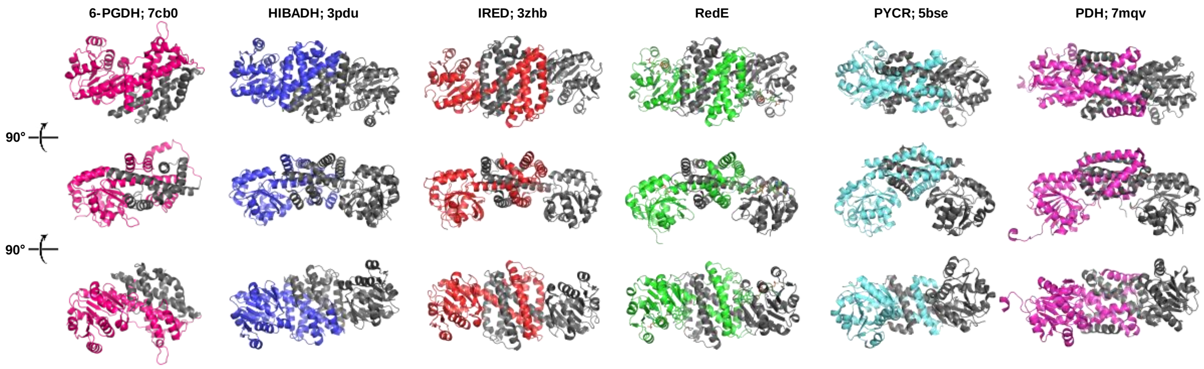

Figure S4. Ninety degree separated views of representative dehydrogenases structurally related to RedE. 6-PGDH is shown as a monomer with the last α-helix involved in dimerization hidden and the C-terminal extension mimicking the βHAD fold homodimer interface coloured grey.

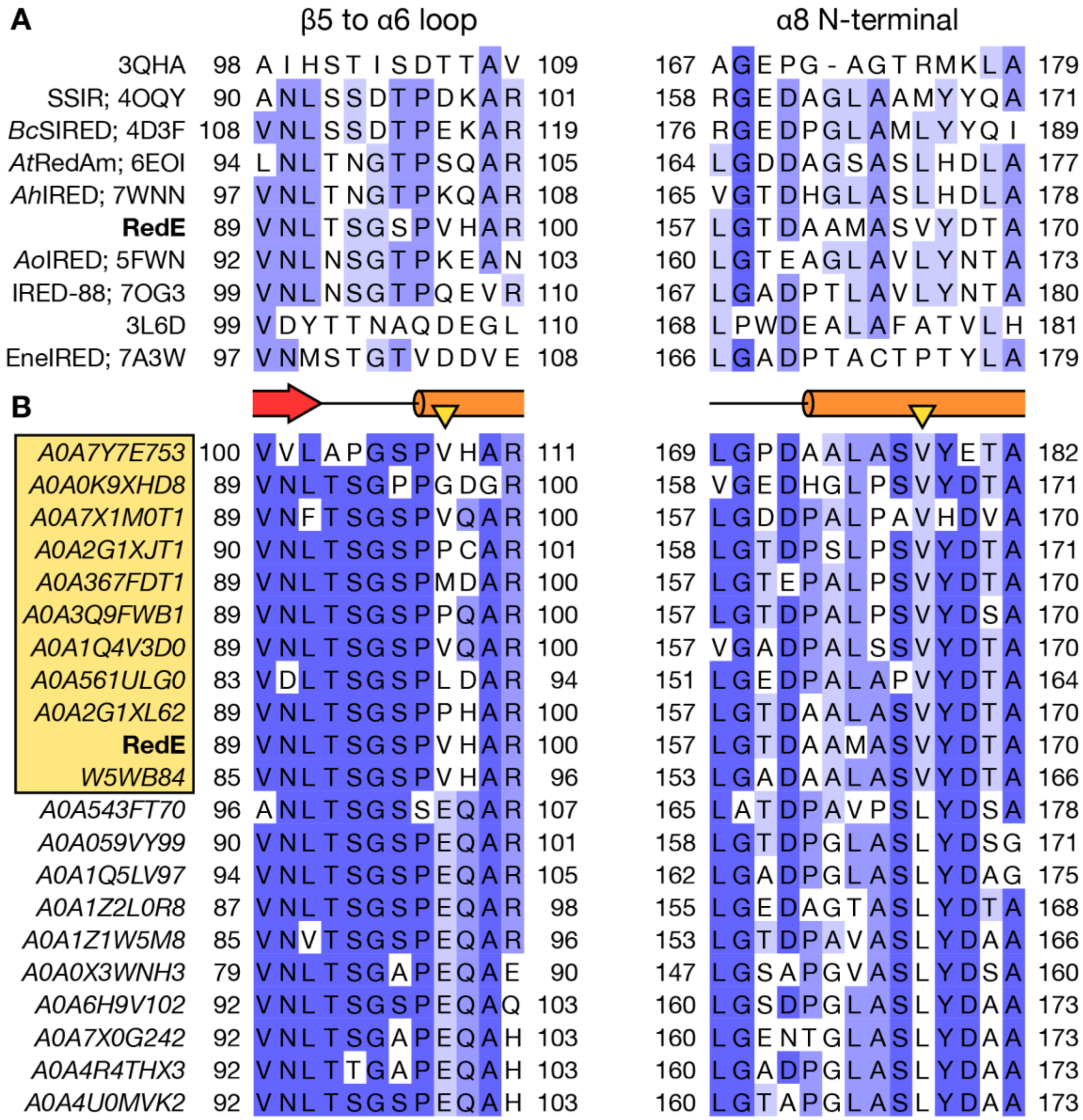

Figure S5. Multiple sequence alignments of residues forming the contours of the secondary binding cleft in RedE featuring the loop between $\beta5$ and $\alpha6$ (left), and the N-terminal region of the interdomain helix ($\alpha8$, right). (A) Nine of sixteen unique IRED or RedAm search hits from secondary structure matching of the RedE protomer. (B) Sequence similarity searches of the UniProt database find ten other IRED-like proteins with a PGGN-like loop (yellow). Each does not have a conserved glutamate residue at V97 (RedE numbering) and preserves a valine residue at V166 rather than a leucine (arrow).

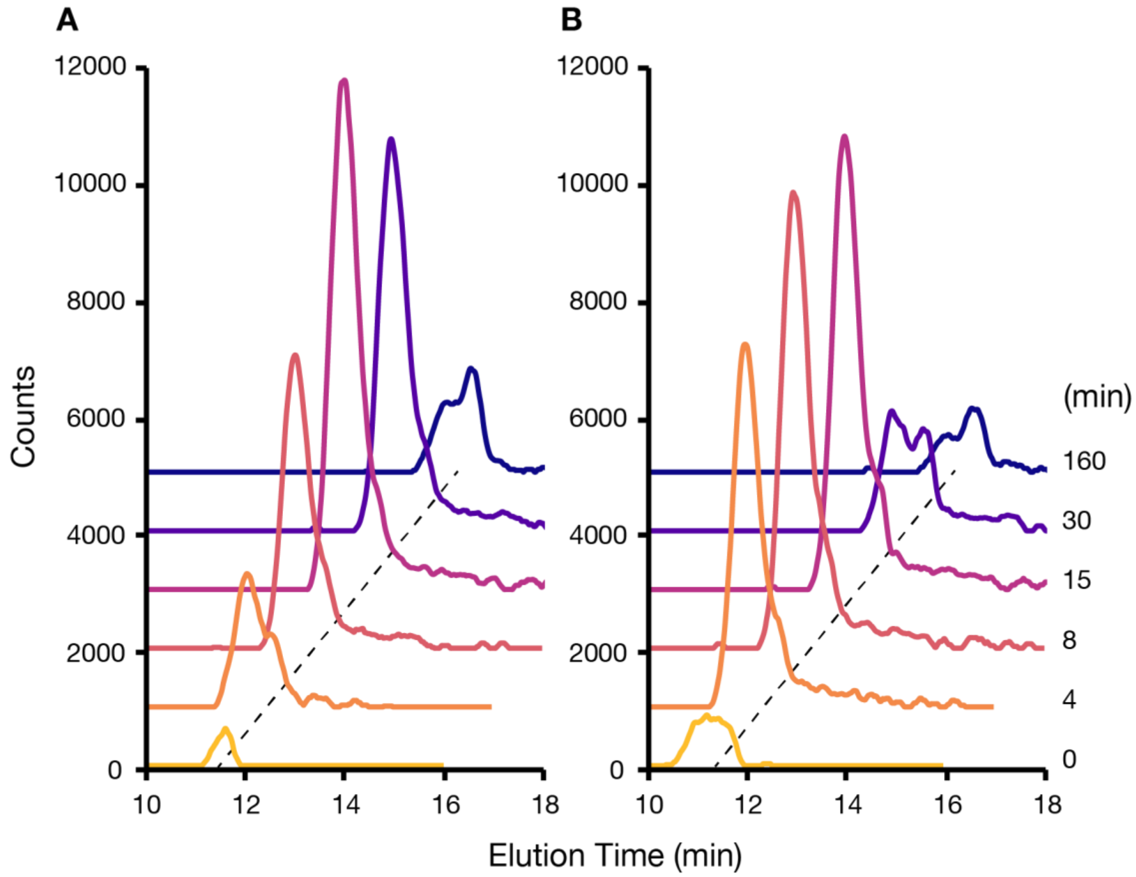

Figure S6. Extracted ion chromatograms for m/z = 296, the mass consistent with indolocarbazole **3**, of StaP reaction products over 2.5 h. (A) Reaction includes 500 μM CPA, 1 mM NADPH, 1 μM StaP, 5 μM FDR, 20 μM FDX, 20 mM HEPES pH 8, and (B) supplemented with 1 mg/ml catalase.

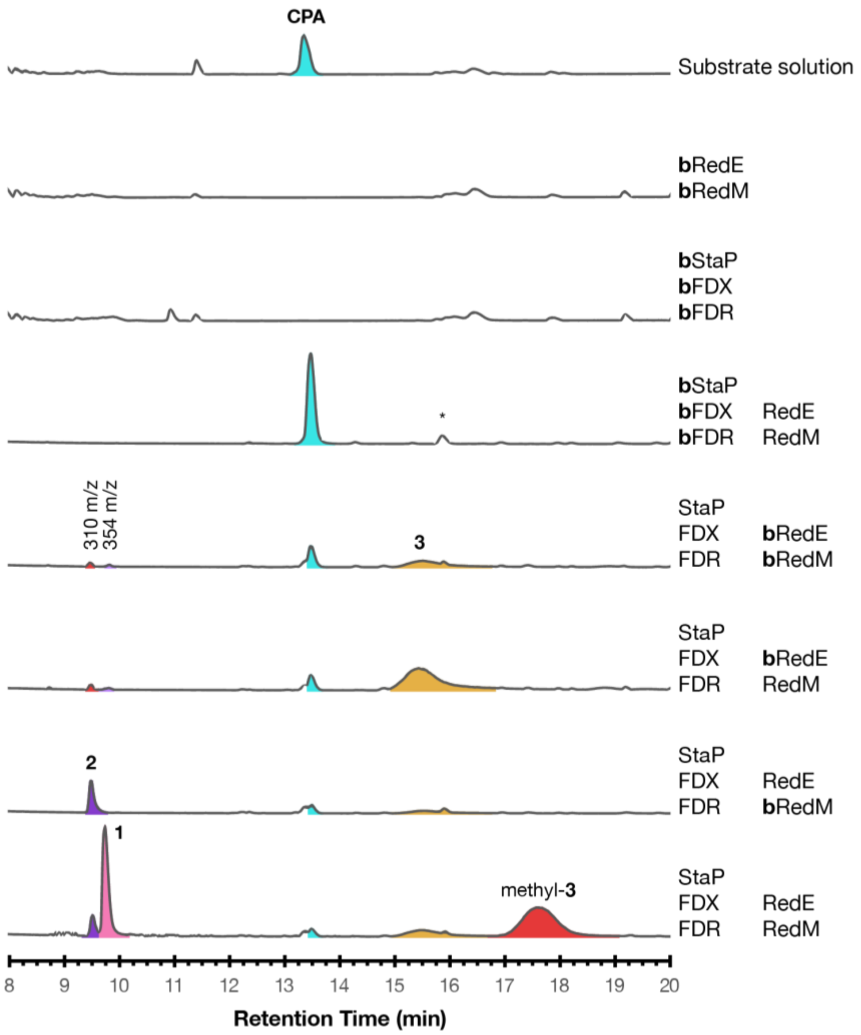

Figure S7. Reaction mixtures of indolocarbazole synthase StaP alongside electron transfer coenzymes ferredoxin and ferredoxin reductase as well as RedE and RedM, each inactivated in turn. Each condition with active StaP accumulates compound **3**, with notable enrichment when RedM is present. Methylated **3** accumulates when both RedE and RedM are active and present. Thus, RedM may not methylate **3** directly. Instead, it may result from non-specific oxidation of methyl-**2** or the reverse reaction of RedE and $NADP^{+}$. Small amounts of indolocarbazoles consistent with 6,7-didehydro-K252c ([M+H]+ = 310.09755 m/z, 0.19 ppm) and 7-carboxy-6,7-didehydro-K252c ($[M+H]^{+}$ = 354.08724 m/z, 0.25 ppm) accumulate when inactivated RedE accompanies active StaP in solution.

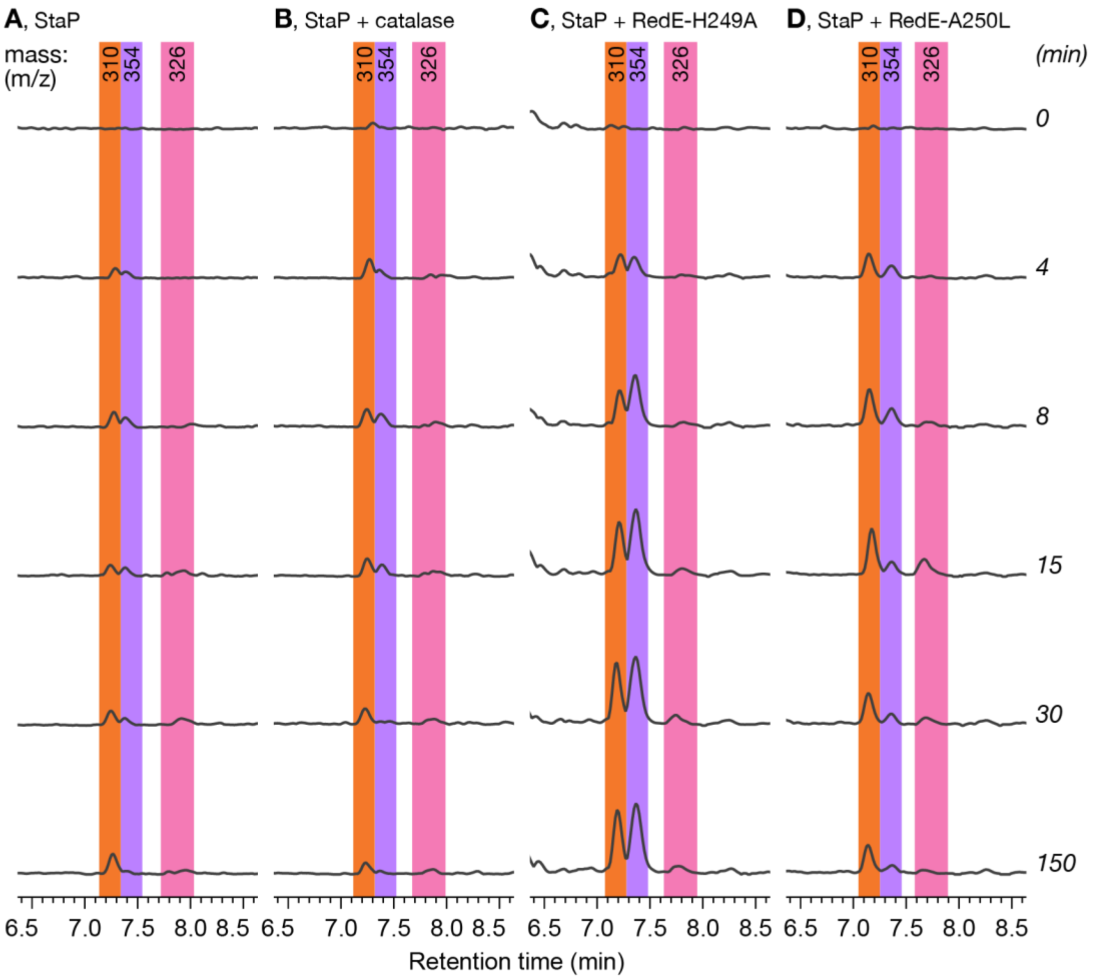

Figure S8. Base peak chromatograms (to scale) of StaP reaction products over 2.5 h. The reaction includes 200 μM CPA, 5 mM NADPH, 1 μM StaP, 1 μM FDR, 20 μM FDX, 20 mM HEPES pH 8, supplemented with (A) water, (B) 1 mg/mL catalase, (C) 20 μM RedE-H249A or (D) 20 μM RedE-A250L. Disrupting the secondary indolocarbazole binding site results in the enrichment of indolocarbazole compounds with masses 310 m/z and 354 m/z found under normal StaP reaction conditions, corresponding with the molecular formulas of 6,7-didehydro-K252c and 7-carboxy-6,7-didehydro-K252c by high-resolution mass spectrometry. The compound with mass 326 m/z accumulates as a result of StaP activity, but does not absorb above 230 nm, typical for indolocarbazoles.

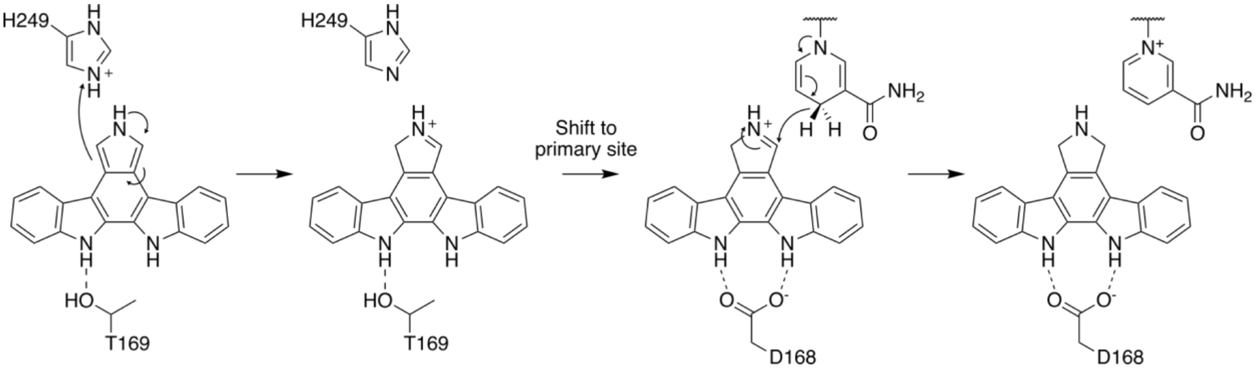

Figure S9. Residue H249 in the secondary binding site potentially selects for the imine tautomer of **3** that lines up for reduction upon direct transfer to the primary active site.

Table S1. The sixteen unique IRED and RedAm hits from secondary structure matching of RedE using PDBeFOLD.

Representative PDB ID	UniProt ID	RMSD (Å)	Percent aligned C$_\alpha$	Percent identity
5A9S	R4SNK4	2.00	91.7%	43.7%
5OCM	D2B7Z8	2.15	95.7%	51.1%
3ZHB	Q1EQE0	2.17	94.4%	46.8%
3L6D	Q88J51	2.33	93.6%	23.5%
6GRL	I0UYD6	2.60	93.4%	36.3%
5OJL	Q0CCT3	2.66	92.4%	35.8%
4OQZ	S3Z901	2.69	93.4%	28.9%
3QHA	A0A0H2ZSR3	2.71	78.9%	16.0%
4D3F	*Not available*	2.81	94.9%	31.5%
6SLE	Q4WDZ8	2.84	92.3%	32.9%
4OQY	M4ZS15	2.94	88.6%	32.2%
4D3S	A0A0J9X1X6	3.02	90.9%	33.7%
6TO4	L7U9F5	3.13	92.7%	38.2%
6SMT	A0R5X0	3.16	94.8%	33.1%
6JIT	D3Q3R0	3.18	90.0%	33.0%
7OSN	A0A1S8Y2S4	3.74	76.1%	43.1%
**Median**		2.76	92.5%	33.4%
**Average**		2.76	90.9%	35.0%

Table S2. Normalized UV-Vis spectra and high-resolution masses for indolocarbazoles produced in tandem StaP/RedE/RedM assays.

	**2**	demethylreductasporine	reductasporine (**1**)
λ (nm)	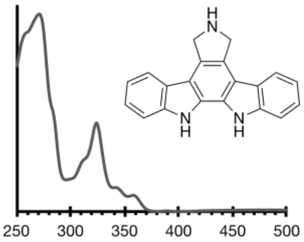	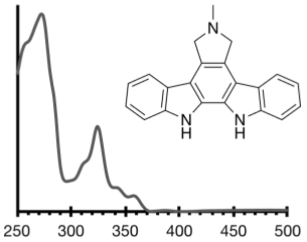	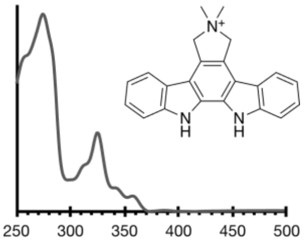
M_{calc} (m/z)	$[M+H]^+$ = 298.13387	$[M+H]^+$ = 312.14952	$[M]^+$ = 326.16517
M_{obs} (m/z)	$[M+H]^+$ = 298.13405 (0.61 ppm)	$[M+H]^+$ = 312.14967 (0.48 ppm)	$[M]^+$ = 326.16540 (0.71 ppm)
	**3**	N-methyl-**3**	6,7-dehydro-K252c
λ (nm)	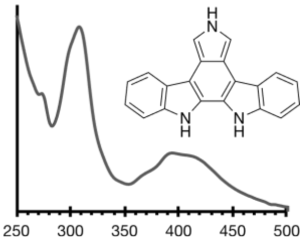	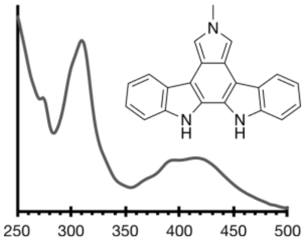	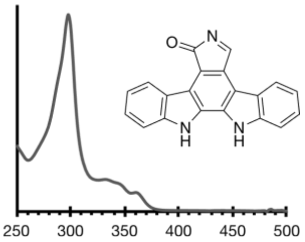
M_{calc} (m/z)	$[M+H]^+$ = 296.11822	$[M+H]^+$ = 310.13387	$[M+H]^+$ = 310.09749
M_{obs} (m/z)	$[M+H]^+$ = 296.11825 (0.10 ppm)	$[M+H]^+$ = 310.13397 (0.32 ppm)	$[M+H]^+$ = 310.09755 (0.19 ppm)
	K252c (**5**)	7-hydroxy-K252c (**6**)	arcyriaflavin A (**AFA**)
λ (nm)	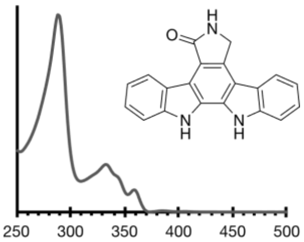	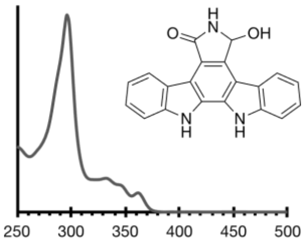	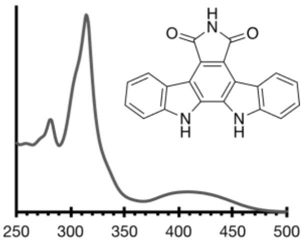
M_{calc} (m/z)	$[M+H]^+$ = 312.11314	$[M+H]^+$ = 328.10805	$[M+H]^+$ = 326.09240
M_{obs} (m/z)	$[M+H]^+$ = 312.11315 (0.03 ppm)	$[M+H]^+$ = 328.10808 (0.09 ppm)	$[M+H]^+$ = 326.09245 (0.16 ppm)
	342 m/z	7-carboxy-6,7-dehydro-K252c	chromopyrrolic acid (**CPA**)
λ (nm)	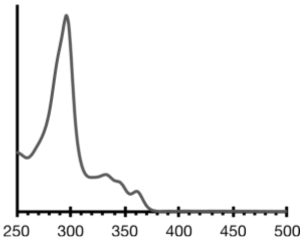	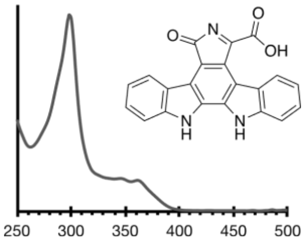	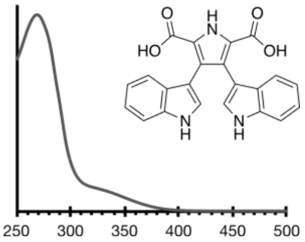
M_{calc} (m/z)		$[M+H]^+$ = 354.08732	$[M+H]^+$ = 386.11353
M_{obs} (m/z)		$[M+H]^+$ = 354.08724 (-0.25 ppm)	$[M+H]^+$ = 386.11366 (0.42 ppm)
